# Distinct lower respiratory tract microbiota profiles linked to airway mucus hypersecretion in children with *Mycoplasma pneumoniae* pneumonia

**DOI:** 10.3389/fmicb.2024.1491506

**Published:** 2024-10-17

**Authors:** Xiwen Wei, Wan Wang, Hang Cheng, Yin Huang, Qixian Zhou, Xiaopeng Yuan

**Affiliations:** ^1^Department of Laboratory Medicine, Zhujiang Hospital, Southern Medical University, Guangzhou, China; ^2^Department of Laboratory Medicine, The Affiliated Foshan Women and Children Hospital, Guangdong Medical University, Foshan, China; ^3^Department of Laboratory Medicine, Shenzhen People’s Hospital (The Second Clinical Medical College, Jinan University, The First Affiliated Hospital, Southern University of Science and Technology), Shenzhen, China; ^4^Department of Pediatrics, The Affiliated Foshan Women and Children Hospital, Guangdong Medical University, Foshan, China

**Keywords:** *Mycoplasma pneumoniae* pneumonia, lower respiratory tract microbiota, airway mucus hypersecretion, co-infection, children

## Abstract

**Background:**

Airway mucus hypersecretion (AMH) can occur in children with acute respiratory diseases, but its underlying mechanisms and relationship with the lower respiratory tract microbiota (LRTM) are not yet fully understood. This study investigates the characteristics of LRTM in children with *Mycoplasma pneumoniae* pneumonia (MPP) and its impact on AMH.

**Methods:**

We collected bronchoalveolar lavage fluid and related clinical indicators from 202 children with MPP. 16S rRNA gene amplicon sequencing was used for detection and identification. Microbial diversity and characteristic genera were compared, and their abundance was analyzed for correlations with clinical factors.

**Results:**

As the disease course (days from onset to bronchoscopy, grouped into T1, T2, T3) extended, α-diversity of the LRTM gradually increased, particularly in the T3 hypersecretion group. Moreover, significant differences were observed in the incidence of AMH, co-infection rates, peripheral white blood cell (WBC) count, and C-reactive protein levels. In AMH, *Mycoplasmoides* and *Veillonella* abundance and peripheral neutrophils were risk factors for increased secretions. In addition, in the T3 co-infection group, *Streptococcus* and *Prevotella* increased, replacing *Stenotrophomonas* as the dominant genus, possibly due to β-lactam antibiotic use. *Prevotella* abundance was strongly correlated with WBC.

**Conclusion:**

The composition and structure of LRTM in children with MPP played a crucial role in AMH and disease progression.

## 1 Introduction

*Mycoplasma pneumoniae* (*M. pneumoniae*) is a common pathogen responsible for community-acquired pneumonia in children (Wang et al., 2022). In recent years, there was an increase in the incidence of *Mycoplasma pneumoniae* pneumonia (MPP), with more than 50% of children admitted to respiratory wards having been diagnosed with MPP (Yan et al., 2024). The known pathogenic mechanisms of *M. pneumoniae* infection include adhesion, oxidative stress, community-acquired respiratory distress syndrome (CARDS) toxin, and immune-mediated inflammatory damage, all of which have the potential to lead to airway mucus hypersecretion (AMH) (Yao and Han, 2021). Excessive airway mucus secretion not only produces highly viscous and elastic mucus, which is difficult to remove, leading to mucus retention, even mucus plug formation. This subsequently exacerbates infections, causes airway obstruction, and airflow limitation, ultimately resulting in progressive decline in lung function (Bao and Zhang, 2018).

In children with MPP, AMH may be closely associated with the imbalance of lower respiratory tract microbiota (LRTM). Studies showed that bronchoscopy was one of the methods for evaluating airway mucus secretion, and the bronchoscopy mucus score system (BS score) for children quantified the amount and distribution of airway mucus (Chang et al., 2006; Xia and Luo, 2022). Moreover, studies on pediatric non-cystic fibrosis bronchiectasis demonstrated that the microbial growth rate in bronchoalveolar lavage fluid (BALF) increased with higher BS scores (Nursoy et al., 2021).

The LRTM not only plays a crucial role in maintaining respiratory homeostasis but may also influence the onset and progression of respiratory diseases through interactions with the host (Wang et al., 2023). Under certain conditions, bacteria that are normally symbiotic can become involved in pathogenic processes, triggering inflammatory responses (Kim et al., 2016). Conversely, pulmonary microbiota may suppress other pathogenic microorganisms (Wu and Segal, 2018) by competing for nutrients (Spiga et al., 2023) or producing metabolites (Hardy and Merrell, 2021). Changes in the structure and composition of these microbial communities may affect mucus secretion in the airways, thereby influencing the course and prognosis of MPP.

However, studies on the correlation between LRTM and AMH remain limited. Therefore, this study employed 16S rRNA amplicon sequencing to investigate the structure and compositional trends of LRTM under varying mucus secretion states in children with MPP. We analyzed the impact of characteristic bacterial genera on AMH to provide new insights and evidence for clinical interventions.

## 2 Materials and methods

### 2.1 Study population and sample collection

In this cross-sectional study, we included pediatric patients who had been hospitalized for MPP at the Foshan Maternal and Child Health Hospital between October 2023 and January 2024. Electronic bronchoscopy (EOB) was performed on these patients, and BALF was collected for subsequent 16S rRNA sequencing. Additionally, demographic characteristics, clinical features, laboratory results, and imaging results were documented. The following criteria were used to include participants in the study: (1) A diagnosis of pneumonia in accordance with the “Guidelines for Diagnosis and Treatment of *Mycoplasma pneumoniae* Pneumonia in Children (2023 Edition)”(National Health Commission of the People’s Republic of China, 2023); (2) The age of the children was ≤13 years; (3) The children underwent EOB during their period of hospitalization. The following criteria were used to exclude participants from the study: (1) Children with chronic respiratory diseases, and those whose duration from disease onset to EOB exceeded one month; (2) Those with severe diseases such as heart, liver, kidney diseases, or tumors; (3) Those with congenital diseases such as congenital heart disease; (4) Those with epilepsy or other mental disorders; (5) Cases without BS scoring under bronchoscopy; (6) Cases with insufficient sample volume to extract respiratory microbiota DNA or failure to construct a sequencing library.

In accordance with the BS scoring system and as detailed in the studies (Chang et al., 2006; Cai et al., 2023) on airway mucus hypersecretion, BS scores of 1–3 entered into the non-hypersecretion group, and scores of 4–6 entered the hypersecretion group. The pre-EOB period refers to the time from illness onset to the day before EOB. The co-infection refers to the simultaneous infection of children with *M. pneumoniae* and other pathogens.

With the consent of the patient’s guardian, one chest CT or X-ray was performed before admission or bronchoscopy. Additionally, a follow-up chest X-ray was conducted 3 to 5 days after bronchoscopy to assess the resolution of lung inflammation. Oxygen therapy was administered to children with pulse oximetry levels below 95%. In the cases we collected, almost all children received nebulized corticosteroid treatment, such as budesonide suspension, prior to bronchoscopy. Therefore, this study focused more on the duration of oral and intravenous corticosteroid use, such as methylprednisolone sodium succinate. According to the guidelines (National Health Commission of the People’s Republic of China, 2023), for children with a confirmed diagnosis of mild MPP, we administered oral or intravenous azithromycin at a dose of 10 mg/kg/day for 3–5 days per course, followed by a second course after an interval of 3–4 days. For severe MPP, intravenous azithromycin was administered at 10 mg/kg/day for 7 consecutive days, along with routine administration of methylprednisolone at 2 mg/kg/day. For children with a drug reaction to azithromycin, erythromycin was used at a dose of 30–45 mg/kg/day for 10–14 days per course. The cases included patients recently referred from external hospitals after unsuccessful treatment. They met the inclusion and exclusion criteria and had detailed diagnostic and treatment records.

BALF should be collected from pediatric patients meeting the specified criteria, in quantities of 2–4 mL. The sample should then be placed in a sterile centrifuge tube and centrifuged at 15,600 × g at 4°C for 30 min. The supernatant should be discarded, and the pellet should be stored immediately at −80°C for future use in 16S rRNA gene amplicon sequencing.

### 2.2 DNA extraction and 16S rRNA gene sequencing

The total bacterial DNA in BALF was extracted using the cetyltrimethylammonium bromide (CTAB) method. The concentration and quality of DNA were evaluated through the use of 2% agarose gel electrophoresis. Following the completion of quality checks on the DNA samples, amplification of the V4 hypervariable region of the bacterial 16S rRNA gene was conducted using universal primers: forward primer 515F and reverse primer 806R. Paired-end sequencing was conducted on the Illumina NovaSeq-PE250 platform. Raw sequencing data were processed with QIIME2 (version 2023.9.1). Demultiplexing and quality inspection of paired-end reads were performed using the “demux” plugin, while trimming of Illumina adapter sequence was performed using the “cutadapt trim-paired” plugin. Denoising of paired-end reads was performed using the “dada2 denoised-paired” plugin, which allowed to adjust the number of 5′- and 3′-end trimmed bases to achieve a read length of 250 bases, followed by filtration and deionization to remove primer sequences or low-quality sequences (minimum quality score of 30). This led to an approximately 70% good-merged reads output. Amplicon sequence variants (ASVs) were assembled using the “feature-table summarize” plugin, while the taxonomy annotation was classified by the pretrained naive Bayes classifier based on the Greengenes database (version 2022.10) with an identity cutoff of 99%.

### 2.3 Data processing and statistical analysis

Alpha diversity was estimated using the Shannon index; while beta diversity was calculated using weighted-UniFrac distances and sample clustering was visualized with Principal Component Analysis (PCA) plots. The online CMAP Microbial Analysis Platform^1^ was utilized. The organization and statistical analysis of the data were conducted using Zstats 1.0^2^ and R version 4.4.0.

Continuous variables were described using median and interquartile range (IQR), and comparisons between groups were made using Mann–Whitney *U* test or Kruskal–Wallis rank-sum tests, as appropriate. Categorical variables were presented as frequencies and percentages and analyzed using the chi-square test or Fisher’s exact test, as appropriate. A multivariate binary logistic regression model was employed to examine the relationship between the predictor variables and the outcomes variables. All statistical tests were conducted with a two-sided hypothesis and a significance level of *p* < 0.05.

## 3 Results

### 3.1 Increased LRTM diversity with the extended pre-EOB periods in AMH

The study included 230 children with MPP following the exclusion of 28 cases due to the presence of other medical histories or interfering factors, the remaining 202 cases were included in the analysis. Amongst these cases, there were 90 males and 112 females, with 97 cases assigned to the hypersecretion group and 105 cases assigned to the non-hypersecretion group. The median of the pre-EOB period was 9 days, with a minimum of 4 days and a maximum of 24 days. Based on tertiles, patients were grouped as follows: T1 (≤7 days), T2 (8–10 days), and T3 (≥11 days).

**Figure 1:**
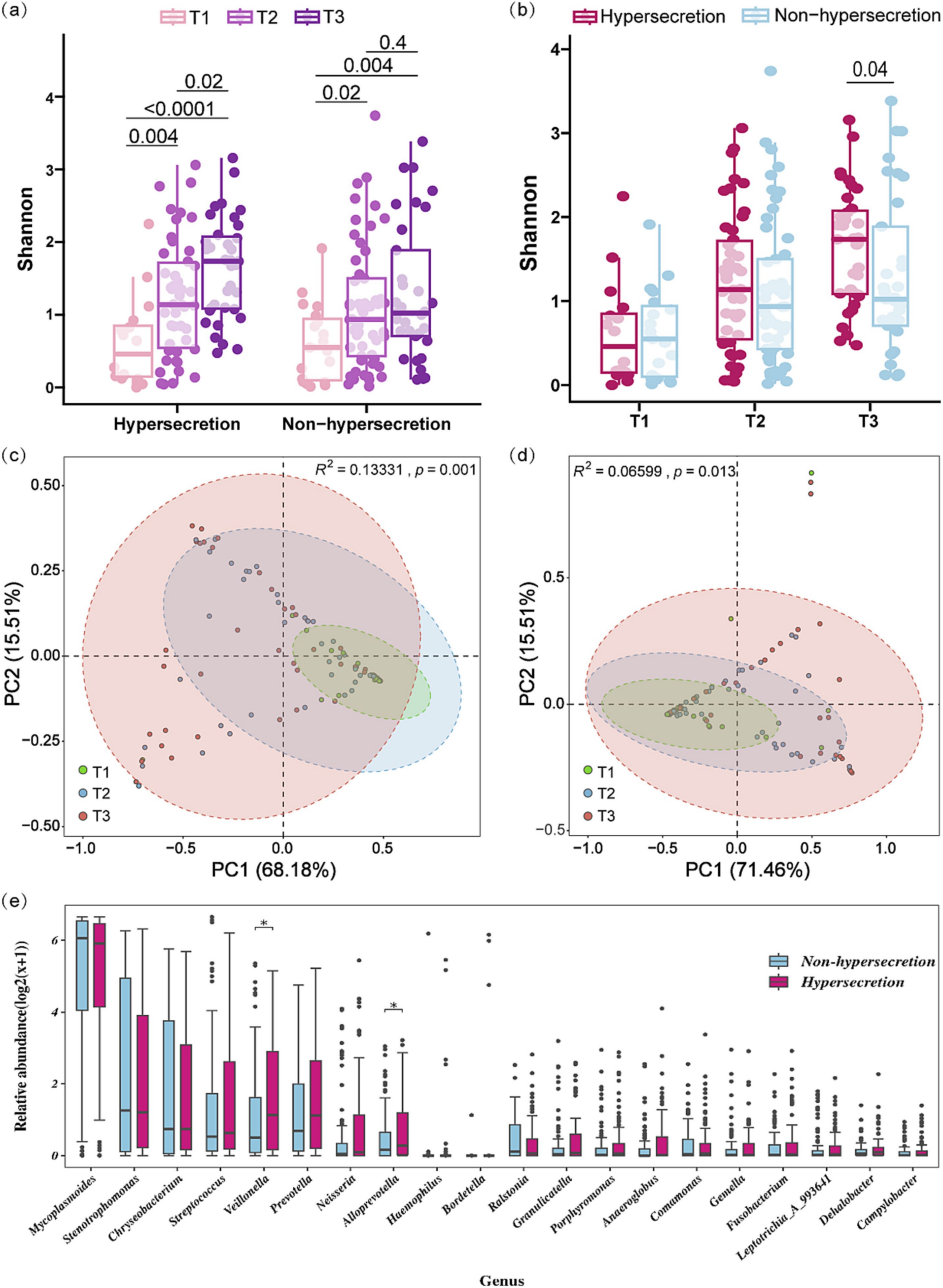
Microbial diversity characteristics across different pre-EOB periods in the hypersecretion and non-hypersecretion groups. (a,b) Multi-group comparison of Shannon index between the hypersecretion and non-hypersecretion groups at different pre-EOB periods. Differences were assessed by Mann–Whitney *U* test with significant *p*-values shown. (c) PCA based on weighted-UniFrac distance among the three pre-EOB periods in the hypersecretion group (T1 = 12, T2 = 45, T3 = 40). Significant differences assessed by Adonis. (d) PCA based on weighted-UniFrac distance among the three pre-EOB periods in the non-hypersecretion group (T1 = 23, T2 = 54, T3 = 28). Differences assessed by Adonis. (e) Differences in the top 20 genera abundance between the hypersecretion (*n* = 97) and non-hypersecretion groups (*n* = 105) were assessed by Mann–Whitney *U* test. *p < 0.05.

16S rRNA amplicon sequencing was conducted on BALF samples, yielding a total of 23,678,356 high-quality tags. Following normalization to the lowest sequences count (42,017), 10,420,216 sequences (51.62%) and 4,394 ASVs were obtained. Subsequently, a total of 59 phyla and 1,264 genera of microbiota were identified. A comparison of microbial diversity between the hypersecretion and *non-hype*rsecretion groups at different pre-EOB periods showed distinct changes (Figure 1). The alpha diversity of both groups exhibited a gradual increase with the pre-EOB period progression, with a notably greater increase observed in the hypersecretion group (Figure 1a). The Shannon index demonstrated a notable increase from 0.459 (0.131, 0.898) at the T1 period to 1.735 (1.076, 2.099) at the T3 period, representing a nearly fourfold expansion. By the T3 period, the alpha diversity in the hypersecretion group was significantly higher than that of the non-hypersecretion group (Figure 1b), with the latter having a Shannon index of only 1.022 (0.674, 2.030). However, in the non-hypersecretion group, there was an increase in alpha diversity from 0.549 (0.080, 0.986) at the T1 period to 0.936 (0.411, 1.527) at the T2 period. This remained relatively stable at the T3 period, with no significant increase observed (Figure 1b).

The results of the PCA analysis indicate significant differences in the microbial community structure across the three pre-EOB periods in both the hypersecretion group (Adonis *R^2^* = 0.133, *p* = 0.001) and the non-hypersecretion group (Adonis *R^2^* = 0.066, *p* = 0.013). At the T1 period, the microbial composition was relatively concentrated. As the disease progressed, inter-individual variation increased, with microbial structure distribution being most dispersed during the T3 period (Figures 1c,d). In the PCA analysis of the hypersecretion group, PC1 and PC2 explained 83.69% of the total variance, indicating that these components effectively represent the data’s variation. At T1, microbial communities were mainly distributed in the positive direction of PC1, reflecting higher diversity and dominance of certain species. In the non-hypersecretion group, T1 communities were primarily in the negative direction of PC1, suggesting a simpler structure with more pathogens lacking competitive advantage. Adonis analysis in this group showed lower explanatory power for microbial differences, but significant changes were still observed.

A comparison of the top 20 genera by relative abundance revealed significantly higher levels of *Veillonella* (1.137 vs. 0.500, *p* = 0.037) and *Alloprevotella* (0.277 vs. 0.159, *p* = 0.022) in the hypersecretion group (Figure 1e). Comparison of the top 10 genera across the three periods showed significant abundance changes, with *Veillonella* notably different (Supplementary Figure S1).

### 3.2 Increased incidence of AMH with the extended pre-EOB periods

**Figure 2:**
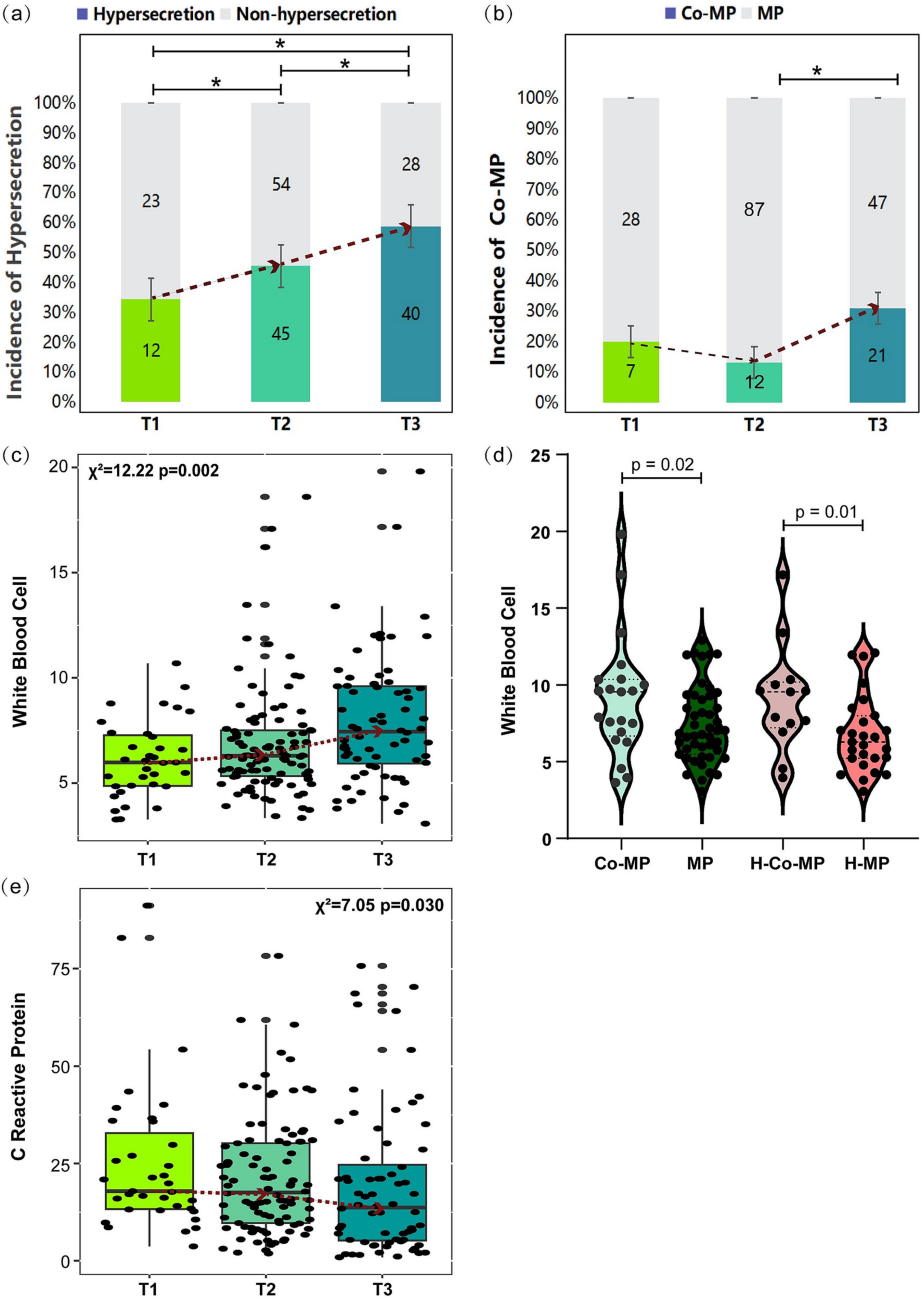
Comparison of selected clinical indicators across pre-EOB periods. (a,b) Incidence of mucus hypersecretion and co-infection across pre-EOB periods. Differences were assessed by chi-square test (*χ*^2^). The dashed line represents the median trend and *p < 0.05 shown T1 (*n* = 35), T2 (*n* = 99), T3 (*n* = 68). (c,e) Changes in WBC and CRP across different pre-EOB periods were assessed by Kruskal–Wallis test (d) Changes in WBC between children with co-infection and only *M. pneumoniae* infection were assessed by Mann–Whitney *U* test. Co-MP (*n* = 40), cooperative infection of *M. pneumoniae* with other pathogens; MP (*n* = 162), *M. pneumoniae* only; H-Co-MP (*n* = 22), cooperative infection of *M. pneumoniae* with other pathogens in hypersecretion group; H-MP (*n* = 75), *M. pneumoniae* in hypersecretion group. WBC, peripheral white blood cells count; CRP, C reactive protein.

Analysis of clinical characteristics across the three pre-EOB groups (Table 1) revealed a notable increase in the incidence of AMH as the disease progresses (*p* = 0.048). This phenomenon reached its peak at the T3 period, where approximately 60% of children experience AMH (Figure 2a). Furthermore, the prevalence of the co-infection was highest during the T3 period (*p* = 0.020), reaching 30% (Figure 2b). This suggests that the probability of co-infection with other pathogens increases with disease progresses.

**Table 1:** Clinical characteristics and differences of MPP across the three pre-EOB periods.

Variables	T1 (*n* = 35)	T2 (*n* = 99)	T3 (*n* = 68)	Statistic	*p*
Age (years), *n* (%)				*χ*^2^ = 2.07	0.355
<7	13 (37.14)	49 (49.49)	35 (51.47)		
≥7	22 (62.86)	50 (50.51)	33 (48.53)		
Peak body temperature (°C)	40.00 (39.40, 40.20)	39.70 (39.30, 40.00)	39.50 (39.00, 40.00)	*χ*^2^ = 8.19^#^	**0.017**
Duration of fever (days)	6.00 (5.00, 7.00)	7.00 (6.00, 7.50)	8.00 (5.00, 10.00)	*χ*^2^ = 15.77^#^	**<0.001**
Macrolides (days)	3.00 (2.00, 4.50)	4.00 (3.00, 6.00)	6.00 (4.75, 7.00)	*χ*^2^ = 32.51^#^	**<0.001**
β-lactams (days)	1.00 (0.00, 3.00)	2.00 (0.00, 3.00)	2.00 (0.00, 5.00)	*χ*^2^ = 2.36^#^	0.308
GCs (days)	0.00 (0.00, 1.00)	0.00 (0.00, 1.00)	0.00 (0.00, 1.00)	*χ*^2^ = 2.88^#^	0.237
NE%	58.4 (49.10, 66.90)	62.40 (56.40, 69.15)	60.75 (51.63, 72.00)	*χ*^2^ = 1.08^#^	0.584
LDH (U/L)	290.00 (250.50, 356.50)	298.00 (260.00, 344.50)	319.50 (290.25, 371.50)	*χ*^2^ = 5.06^#^	0.080
Mucus plug, *n* (%)				*χ*^2^ = 0.71	0.701
No	31 (88.57)	87 (87.88)	57 (83.82)		
Yes	4 (11.43)	12 (12.12)	11 (16.18)		
Tree-in-bud sign, *n* (%)				*χ*^2^ = 1.02	0.600
No	15 (66.67)	41 (70.21)	21 (78.95)		
Yes	15 (33.33)	44 (29.79)	31 (21.05)		
Pleural effusion, *n* (%)				*χ*^2^ = 0.54	0.765
No	29 (87.88)	79 (83.16)	51 (82.26)		
Yes	4 (12.12)	16 (16.84)	11 (17.74)		

Quantitative skewness data are presented as the median (percentile: P25, P75), and categorical data are expressed as the frequency. Macrolides, preoperative macrolide antibiotic days; β-lactams, preoperative β-lactams antibiotic days; GCs, preoperative glucocorticoid days; NE%, percentage of peripheral neutrophils; LDH, lactate dehydrogenase; ALT, alanine aminotransferase; Co-MP, cooperative infection of *M. pneumoniae* with other pathogens.^#^Kruskal-Waills test. The bold values highlight *P* < 0.05, indicating statistically significant differences.

The three pre-EOB periods exhibited significant differences in peak fever (*p* = 0.017), duration of fever (*p* < 0.001), and days of macrolide antibiotics use (*p* < 0.001) (Table 1). It was noteworthy that demonstrate a gradual increase in WBC with disease progresses (Figure 2c), reaching a peak at the T3 period. The WBC was significantly higher in children with co-infection than in those with single *M. pneumoniae* infection (*p* = 0.02), particularly in the hypersecretion group (*p* = 0.01) (Figure 2d). A significant decrease in CRP levels was observed between the T2 and T3 periods (*p* = 0.030) (Figure 2e).

### 3.3 Risk factors for increased mucus secretion: *Mycoplasmoides, Veillonella,* and neutrophil percentage

From the previous analysis, it was evident that microbial diversity is relatively higher in the hypersecretion group. We further analyzed the differences in genera and clinical indicators across different BS scores within the hypersecretion group to illustrate the impact of microbiota on mucus production. Due to having only one case with BS 6, it was excluded for better comparison. We performed a multivariate logistic regression analysis for BS scores 4 and 5, focusing on lung characteristics, inflammation indicators, and differential genera, with *p*-values adjusted for age, gender, and days of macrolide, β-lactam, and glucocorticoid use. As shown in Table 2, NE% (OR 1.07, 95% CI 1.01–1.13, *p* = 0.032), *Mycoplasmoides* (OR 1.03, 95% CI 1.01–1.06, *p* = 0.042), and *Veillonella* abundance (OR 1.11, 95% CI 1.01–1.23, *p* = 0.037) were identified as risk factors for increased mucus secretion. Interestingly, pleural effusion was negatively correlated with BS score 5 (OR 0.09, 95% CI 0.01–0.82, *p* = 0.033).

**Table 2:** Multivariate logistic regression analysis of BS score.

Variables	*β*	*p*	OR (95% CI)
Pre EOB period			
T1 (*n* = 12)			1.00 (Reference)
T2 (*n* = 44)	1.36	0.151	3.91 (0.61–25.15)
T3 (*n* = 40)	2.10	0.069	8.20 (0.85–78.99)
Mucus plug			
No			1.00 (Reference)
Yes	0.54	0.581	1.72 (0.25–11.74)
Pleural effusion			
No			1.00 (Reference)
Yes	−2.46	**0.033**	0.09 (0.01–0.82)
Tree-in-bud sign			
No			1.00 (Reference)
Yes	0.25	0.700	1.29 (0.36–4.62)
NE%	0.06	**0.032**	1.07 (1.01–1.13)
*Mycoplasmoides*	0.03	**0.042**	1.03 (1.01–1.06)
*Veillonella*	0.11	**0.037**	1.11 (1.01–1.23)
Shannon index	−0.86	0.192	0.42 (0.12–1.54)

OR, odds ratio; CI, confidence interval; Adjust: age (month), gender, macrolides, β-lactams, glucocorticoid.The bold values highlight *P* < 0.05, indicating statistically significant differences.

### 3.4 Shift of dominant LRT genera to *Stenotrophomonas* and *Chryseobacterium* in mucus hypersecretion with the extended pre-EOB periods

**Figure 3:**
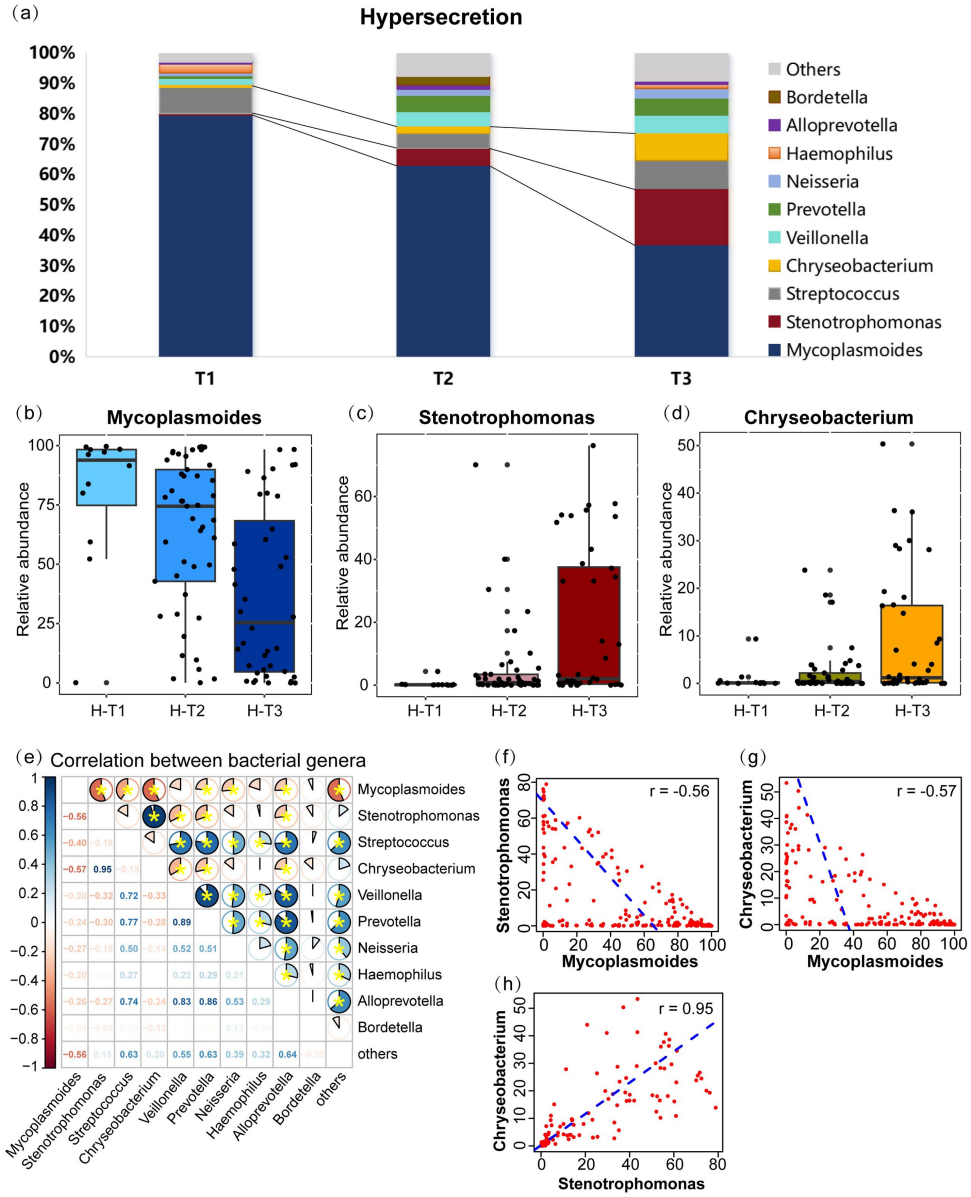
Microbial communities changes and inter-genus correlations in the hypersecretion group as the pre-EOB period progresses. (a) The relative abundances of the top 10 bacterial genera are presented in a stacked bar chart across the three pre-EOB periods T1 (*n* = 12), T2 (*n* = 45), T3 (*n* = 40). (b–d) Changes in the relative abundance of *Mycoplasmoides*, *Stenotrophomonas*, *Chryseobacterium* with the pre-EOB periods. (e) Inter-genus correlations among the top 10 genera by abundance. The pie chart slices represent the correlation and yellow * indicates *p* < 0.05. (f–h) The linear fit graph showing the Spearman correlations between *Mycoplasmoides*, *Stenotrophomonas*, *Chryseobacterium*. H-T1/T2/T3, the three pre-EOB periods in the hypersecretion group.

In the hypersecretion group, the five most prevalent microbial genera was *Mycoplasmoides* (53.3%), *Stenotrophomonas* (12.0%), *Chryseobacterium* (6.5%), *Streptococcus* (5.1%), and *Veillonella* (4.6%), as illustrated in Figure 3a. As the pre-EOB period progresses, there was a notable decline in the relative abundance of *Mycoplasmoides*, from 92.0% in T1 period to 35.3% in T3 period (*p* < 0.001) (Figure 3b). Conversely, the relative abundances of *Stenotrophomonas* (0.2% vs. 3.4%, *p* = 0.009) (Figure 3c) and *Chryseobacterium* (0.1% vs. 2.7%, *p* = 0.007) (Figure 3d) increased. The correlation analysis of the top 10 genera (Figure 3e) indicated a negative correlation between *Mycoplasmoides* and both *Stenotrophomonas* (*r* = −0.56, *p* < 0.05) and *Chryseobacterium* (*r* = −0.57, *p* < 0.05) (Figures 3f,g). In contrast, *Stenotrophomonas* and *Chryseobacterium* was found to be highly positively correlated (*r* = 0.95, *p* < 0.05) (Figure 3h). These findings indicate that as disease course progresses or preoperative antibiotic were administered, the abundance of *Mycoplasmoides* decreased, was replaced by *Stenotrophomonas* and *Chryseobacterium*. These two genera increased synergistically and gradually become the dominant genera in children with MPP. Furthermore, a highly positive correlation was observed between *Prevotella* and other genera, including *Streptococcus* (*r* = 0.77, *p* < 0.05), *Veillonella* (*r* = 0.89, *p* < 0.05), and *Alloprevotella* (*r* = 0.86, *p* < 0.05).

### 3.5 *Prevotella* enrichment and increased WBC in T3 co-infection group with β-lactam use

**Figure 4:**
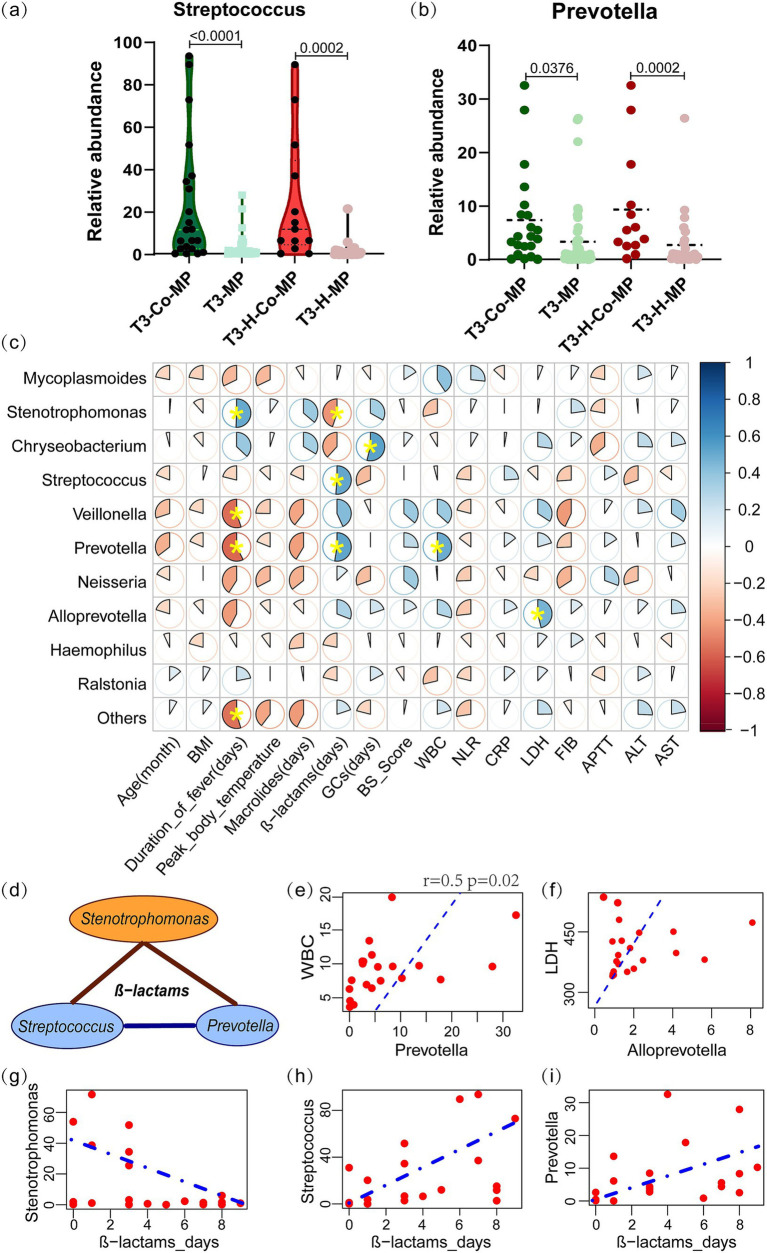
The characteristic microbial communities of co-infection group in the T3 period and their correlation with clinical indicators. (a,b) The *Streptococcus* and *Prevotella* abundances between the co-MP and MP-only groups, as well as within the hypersecretion state, were compared using the Mann–Whitney *U* test. (c) Spearman correlation analysis between the microbial abundance and clinical indicators in T3-co-MP group. The pie chart slices represent the correlation and yellow * indicates *p* < 0.05. (d) Spearman correlation analysis of *Stenotrophomonas* with *Streptococcus* and *Prevotella* under β-lactam antibiotics. The brown line represents a negative correlation, while the blue line represents a positive correlation. (e,f) Linear correlation plots of WBC with *Prevotella*, and LDH with *Alloprevotella*; the dashed line represents the linear fit for the Spearman correlation. (g–i) Linear regression plots showing the correlation between β-lactam antibiotic days and the abundance of *Chryseobacterium*, *Streptococcus*, and *Prevotella*. T3-Co-MP (*n* = 21), co-infection in T3 period; T3-MP (*n* = 47), single *M. pneumoniae* infection in T3 period; T3-H-Co-MP (*n* = 13), co-infection in T3 hypersecretion group; T3-H-MP (*n* = 8), single *M. pneumoniae* infection in T3 hypersecretion group. GCs, preoperative glucocorticoid days; WBC, white blood cells count; NLR, the ratio of neutrophils to lymphocytes; CRP, C reactive protein; LDH, lactate dehydrogenase; ALT, alanine aminotransferase; FIB, fibrinogen; APTT, activated partial thromboplastin time; AST, aspartate aminotransferase.

During the T3 period, children with co-infections had significantly higher abundance of *Streptococcus* (11.75% vs. 0.82%, *p* < 0.001) and *Prevotella* (4.37% vs. 0.88%, *p* < 0.001) compared to those with single *M. pneumoniae* infection, especially in the hypersecretion group (Figures 4a,b). At this point, *Mycoplasmoides* abundance was significantly lower in the co-MP group (*n* = 21), than in the MP group (*n* = 47) (4.18% vs. 49.33%, *p* < 0.001).

Spearman correlation analysis examined the relationship between microbial abundance and clinical indicators in the T3-co-MP group. The analysis revealed a significant positive correlation between *Prevotella* abundance and WBC (*r* = 0.50, *p* = 0.022) (Figures 4c,e), as well as between *Alloprevotella* abundance and LDH (*r* = 0.46, *p* = 0.035) (Figures 4c,f). Additionally, *Prevotella* (*r* = −0.58, *p* = 0.006) and *Veillonella* (*r* = −0.55, *p* = 0.010) were found to be negatively correlated with the duration of fever, while *Stenotrophomonas* (*r* = 0.51, *p* = 0.035) was positively correlated with the duration of fever.

The correlation analysis indicated a significant link between microbial composition and the duration of β-lactam antibiotic use in the T3-co-infection group (Figure 4c). As longer β-lactam antibiotic use, *Stenotrophomonas* (*r* = −0.449, *p* = 0.041) gradually declined (Figures 4d,g) and was replaced by *Streptococcus* (*r* = 0.513, *p* = 0.017) and *Prevotella* (*r* = 0.526, *p* = 0.014) (Figures 4h,i).

## 4 Discussion

Airway mucus hypersecretion has received extensive attention in studies of chronic respiratory diseases such as asthma (Jaramillo et al., 2024), COPD (Tian and Wen, 2015), cystic fibrosis (Mall, 2016), and bronchiectasis (Chen and Zheng, 2021; Abrami et al., 2024). While AMH was also common in acute respiratory infections in children, particularly with infections like *M. pneumoniae*, *Adenovirus* (Cai et al., 2023), *Influenza virus*, respiratory *Syncytial virus* (Yan and Deng, 2024), and *Bordetella pertussis* (Yao and Han, 2021), research on LRTM in children with MPP and mucus hypersecretion is very limited. AMH refered to the pathophysiological process in which various pathogenic factors cause hyperplasia and hypertrophy of airway mucosal goblet cells and submucosal glands, leading to excessive mucus production (Chen and Zheng, 2021). Its severity was related to the quantity and viscosity of airway secretions and the presence of small airway mucus plugs (Chen and Zheng, 2021). This study examined the composition and structural characteristics of the LRTM from two perspectives: disease progression and mucus hypersecretion. We observed that as the pre-EOB period extended, α-diversity gradually increased, particularly in the hypersecretion group. β-diversity indicated significant spatial distribution changes in the microbial community. Importantly, the incidence of AMH increased with disease progression, suggesting a potential relationship between LRTM and AMH, beyond just *M. pneumoniae* infection. Interestingly, the T1 period in the hypersecretion group had the lowest Shannon index, which seems to present a potential contradiction with the sample distribution in the PCA plot at T1. The Shannon index measures species richness and evenness within a sample, reflecting the species diversity of a particular community. PCA analysis measures differences between samples, with a focus on community structure and heterogeneity between samples. The two indexes are complementary to each other.

We focused on analyzing the characteristic genera in AMH. The LRTM of children in T1 hypersecretion group, dominated by *Mycoplasmoides*. *M. pneumoniae* inhibits commensal bacteria by competing for nutrients (Chen et al., 2021) and inducing production of host cytokine, nitric oxide, antimicrobial peptides (Yang et al., 2004). It also invades host cells, depletes nutrients, and releases CARDS toxin, superoxide radicals, and hydrogen peroxide, causing direct epithelial cell damage (Hu et al., 2023). The exotoxin CARDS TX released by *M. pneumoniae* induces “asthma-like” histopathological changes similar to allergic diseases, characterized by elevated eosinophil levels, enhanced Th2 cell responses, increased chemokines expression, increased mucus production, and increased airway resistance (Xu et al., 2024) *M. pneumoniae* can promoting mucin upregulation via STAT6-STAT3 and epidermal growth factor receptor (EGFR) signaling pathways (Hao et al., 2014), and reduces ciliary clearance rate by directly damaging airway epithelial cells (Lu and Zhou, 2018), leading to mucus blockage. *M. pneumoniae* infection increases neutrophil counts, and the neutrophil elastase (NE) released activates the MAPK/ERK pathway and EGFR, stimulating airway epithelial cells to secrete more mucins, particularly MUC5AC and MUC5B (Chen and Tong, 2016). In T1 period, *M. pneumoniae* breaks down glucose through the glycolysis pathway to produce lactate. In T3 period, increased abundances of *Streptococcus* and *Prevotella* provide lactate as the preferred carbon source for the growth of *Veillonella*. *Veillonella* metabolizes lactate into short-chain fatty acids (SCFAs), such as acetate and propionate. It is well known that SCFAs have anti-inflammatory effects. However, studies have also shown that, *in vitro*, propionate in combination with TNF-α can synergistically increase the production of IL-6 and CXCL8 by primary human lung fibroblasts and airway smooth muscle cells (Whiteside et al., 2021). The enrichment of *Streptococcus* and *Veillonella* in airway washings from cancer patients is associated with increased inflammatory cell infiltration and upregulation of the PI3K/ERK signaling pathway in bronchial epithelium (Tsay et al., 2018). *Veillonella* in the oral cavity and respiratory tract forms a multispecies biofilm through symbiosis with late-colonizing pathogens, such as *Streptococcus* (Zhou et al., 2021). *Veillonella* promotes the increase of inflammatory factors through its metabolic products and pro-inflammatory signaling pathways, leading to excessive mucus secretion. The abundance of *Mycoplasmoides* decreased in the T3 period, while *Stenotrophomonas* and *Chryseobacterium* significantly increased. This outcome may result from a combination of microbial interactions, antibiotic use, immune responses, and prolonged disease duration. After all, increased mucin secretion directly suppress pathogen virulence, aiding in microbial coexistence (Wang B. X. et al., 2021). However, the causal relationships require further investigation.

Respiratory microbiota are influenced by migration, elimination, and microbial proliferation rates (Pérez-Cobas et al., 2023). Using a stepwise forward method, we selected clinical indicators and characteristic genera to perform a multivariate logistic regression analysis on BS grades 4 and 5 in the hypersecretion group. The results identified the peripheral neutrophil percentage, *Mycoplasmoides*, and *Veillonella* as risk factors for increased mucus secretion. Studies have shown increased neutrophil infiltration in both peripheral blood and BALF in children with MPP or refractory MPP (Wang B. X. et al., 2021; Cai et al., 2023; Abrami et al., 2024), and that MPP severity is associated with a neutrophil-mediated immune response. In MPP children with mucus plugs, higher serum IFN-γ levels were found in those with pleural effusion, which correlated positively with *Veillonella* abundance (Xu et al., 2020). Overabundance of *Veillonella parvula* induce macrophage-associated inflammation through the LPS-TLR4 pathway, impairing gut motility and further exacerbating gut dysbiosis, which promotes the development of Hirschsprung’s disease-associated enterocolitis (Zhan et al., 2022). These suggest that *Veillonella* may also play a role in disease progression. On the other hand, certain *Prevotella* species, such as *Prevotella timonensis*, have a multitude of predicted α/β-galactosidases, α/β-N-acetylgalactosaminidases, and α/β-N-acetylglucosaminidases, which hydrolyze glycosidic bonds in sialic acid and fucoses. Sialidases and fucosidases are key enzymes that initiate the degradation of O-glycan structures in secreted and epithelial-bound mucins, enabling further mucin degradation after the removal of these outer monosaccharides (Segui-Perez et al., 2024). This plays a regulatory role in mucus production.

Co-infection of *M. pneumoniae* with other pathogens is common in children with respiratory syndromes (Chiu et al., 2015). In this study, 40 co-infection cases were identified through BALF bacterial culture and pathogen DNA testing during the T3 period, with 61.9% of cases involving *Streptococcus pneumoniae*, 19% *Haemophilus influenzae*, 38.1% virus. Co-infection rates were highest in the T3 period, and elevated WBC levels compared to case with only *M. pneumoniae*. Additionally, the abundance of *Streptococcus* and *Prevotella* increased significantly with β-lactam antibiotics use, and *Prevotella* was linearly correlated with WBC. The rise of pathogenic *Streptococcus* and *Prevotella* replaced the previously dominant *Stenotrophomonas*. In certain environments, such as CF lung microbial communities, *Prevotella* has been reported to produce β-lactamase, protecting *P. aeruginosa* from ceftazidime. This leads to antibiotic-resistant strains dominating the microbial community (Vandeplassche et al., 2019). As the disease progresses, the likelihood of co-infection increased. This may be due to airway epithelial damage from *M. pneumoniae* (Zhou et al., 2022; Hu et al., 2023) and the rise of opportunistic pathogens caused by prolonged antibiotic use, leading to microbiota imbalance and increased infection risk (Grice and Segre, 2012; Wang et al., 2024). A study of bronchial mucus plugs revealed that the most prevalent genera in BALF of children with MPP were *Mycoplasmoides*, *Prevotella*, and *Streptococcus* (Xu et al., 2020). Several species of *Prevotella* are known to cause opportunistic infections (Larsen, 2017). Micro-aspiration of *Prevotella* during subclinical airway inflammation may occur (Huffnagle et al., 2017), though the specific mechanism linking *Prevotella* and inflammatory cells remains unclear and requires further study. The dominant genera that increased during the T3 period, *Stenotrophomonas* and *Chryseobacterium*, were also considered common opportunistic pathogens resistant to most β-lactam and macrolide antibiotics (Klimkaitė et al., 2023), showed a strong correlation, suggesting potential synergism. *Stenotrophomonas* was found to utilize various carbon sources, inhibit other microorganisms, and stimulate T lymphocytes to secrete IL-2, IFN-γ, and TNF-α, promoting lymphocyte apoptosis and immune suppression (Wang M. et al., 2021). A 9-year retrospective study found that most *Chryseobacterium indologenes* infections were hospital-acquired. The bacterium was found to colonize and spread via contaminated, moist medical equipment, and its prevalence has increased with the use of broad-spectrum antibiotics and invasive procedures (Zhang et al., 2021). *Veillonella* depends on lactate-producing bacteria such as *Streptococcus* and *Prevotella* for survival, which represents a typical metabolic symbiotic relationship*. Streptococcus pneumoniae i*s one of the common co-infecting species in MPP patients, capable of exacerbating lung inflammation and increasing secretions and mucus production. *Veillonella* secretes catalase to break down the H₂O₂ produced by the metabolism of Streptococcus, providing a stable anaerobic environment that facilitates the growth of oxygen-sensitive genera (Zhou et al., 2017) such as *Prevotella* and *Alloprevotella.* This also explains the positive correlation between *Veillonella* and *Prevotella* and *Alloprevotella*. *Stenotrophomonas* and *Chryseobacterium* are opportunistic pathogens that are resistant to multiple broad-spectrum antibiotics. They coexist under antibiotic use, potentially forming synergistic resistance to antibiotics, which contributes to disease progression and creates competition with other anaerobic genera such as *Veillonella* and *Prevotella* The guidelines (National Health Commission of the People’s Republic of China, 2023) for the diagnosis and treatment of MPP suggest that if macrolide treatment fails to resolve fever within 72 h and clinical signs and chest imaging show no improvement or worsening, it is diagnosed as macrolide unresponsive MPP (MUMPP). In such cases, timely replacement with drugs such as doxycycline is recommended. The aim is timely intervention to reduce the risk of severe disease and complications. However, diagnosing MUMPP is not easy. From our study, it can be observed that in children with MPP, as the disease progresses and antibiotics are used, including macrolides and empirical β-lactams, airway microbial diversity begins to increase after 7 days of infection. After 10 days of infection, under conditions of mucus hypersecretion, both the likelihood of infection with drug-resistant opportunistic pathogens and common pathogens begins to rise, affecting mucus production and disease progression.

In certain environments, such as cystic fibrosis lung microbial communities, *Prevotella* has been reported to produce β-lactamase, protecting *P. aeruginosa* from ceftazidime. This leads to antibiotic-resistant strains dominating the microbial community (Vandeplassche et al., 2019). Among children with T3-period infections (*n* = 21), 13 (13/21) received β-lactam antibiotics for more than 7 days before EOB, with seemingly limited efficacy against Streptococcus. Additionally, 5 (5/21) received glucocorticoid treatment before EOB, with 3 using glucocorticoids for ≤3 days, all of whom experienced recurrent fever. This may be linked to dosage and duration, but also suggests heightened inflammatory responses. *Stenotrophomonas maltophilia*, a pathogen causing hospital-acquired infections, produces cytokines like IL-2, IFN-γ, and TNF-α, triggering systemic inflammation (Wang M. et al., 2021). TNF-α, a key fever mediator, induces fever via the hypothalamic thermoregulatory center, explaining the positive correlation between *Stenotrophomonas* and febrile days. Increased *Stenotrophomonas* abundance in sputum has been linked to poor antibiotic outcomes in acute exacerbations of COPD (Liu, 2020). In our study, *Stenotrophomonas* showed a negative correlation with β-lactam antibiotics, suggesting sensitivity to these drugs. As *Stenotrophomonas* levels declined, *Prevotella* and *Veillonella* abundance increased, with *Veillonella*-produced SCFAs playing an anti-inflammatory role, resulting in fewer febrile days. In later disease stages, microbial diversity increased, complicating the relationship due to antibiotic use, warranting further data analysis.

The complex use of antibiotics before admission can affect the BALF microbiota structure, and there are currently no clinical indicators and methods to correct for these factors. In the future, conducting controlled longitudinal studies with adequate sample sizes and integrating multiomics approaches could provide robust data for comprehensive statistical analyses of antibiotics and the LRTM (Pérez-Cobas et al., 2023). Due to the limitations of 16S rRNA sequencing, higher-resolution metagenomic sequencing is planned for the next phase to explore the mechanisms by which bacterial species (Bars-Cortina et al., 2024) in the LRT influence mucus secretion and host immunity.

In conclusion, we analyzed the diversity of LRTM in children with MPP from two perspectives: disease progression and mucus hypersecretion. Longer disease courses showed richer diversity, especially in the AMH conditions. We identified *Mycoplasmoides*, and *Veillonella*, along with peripheral neutrophils, as risk factors for increased mucus production. In co-infected MPP, *Streptococcus* and *Prevotella* were dominant in the LRTM, particularly under AMH conditions, and were significantly positively correlated with β-lactam antibiotic use. *Prevotella* was also positively correlated with WBC during this period. These suggested that characteristic genera in the lower respiratory tract of MPP children played a crucial role in influencing AMH and disease progression.

## Data Availability

The datasets presented in this study can be found in online repositories. The names of the repository/repositories and accession number(s) can be found here: https://ngdc.cncb.ac.cn/gsa/, PRJCA029662.

## Footnotes

^1^http://www.cmap-zjyy.com:10548/app/cmap

^2^www.zstats.net
