# Supplementary figures and images for: Distinct lower respiratory tract microbiota profiles linked to airway mucus hypersecretion in children with Mycoplasma pneumoniae pneumonia
Source: Front Microbiol. 2024 Oct 17;15:1491506. doi: 10.3389/fmicb.2024.1491506 (PMC11524823; doi:10.3389/fmicb.2024.1491506)

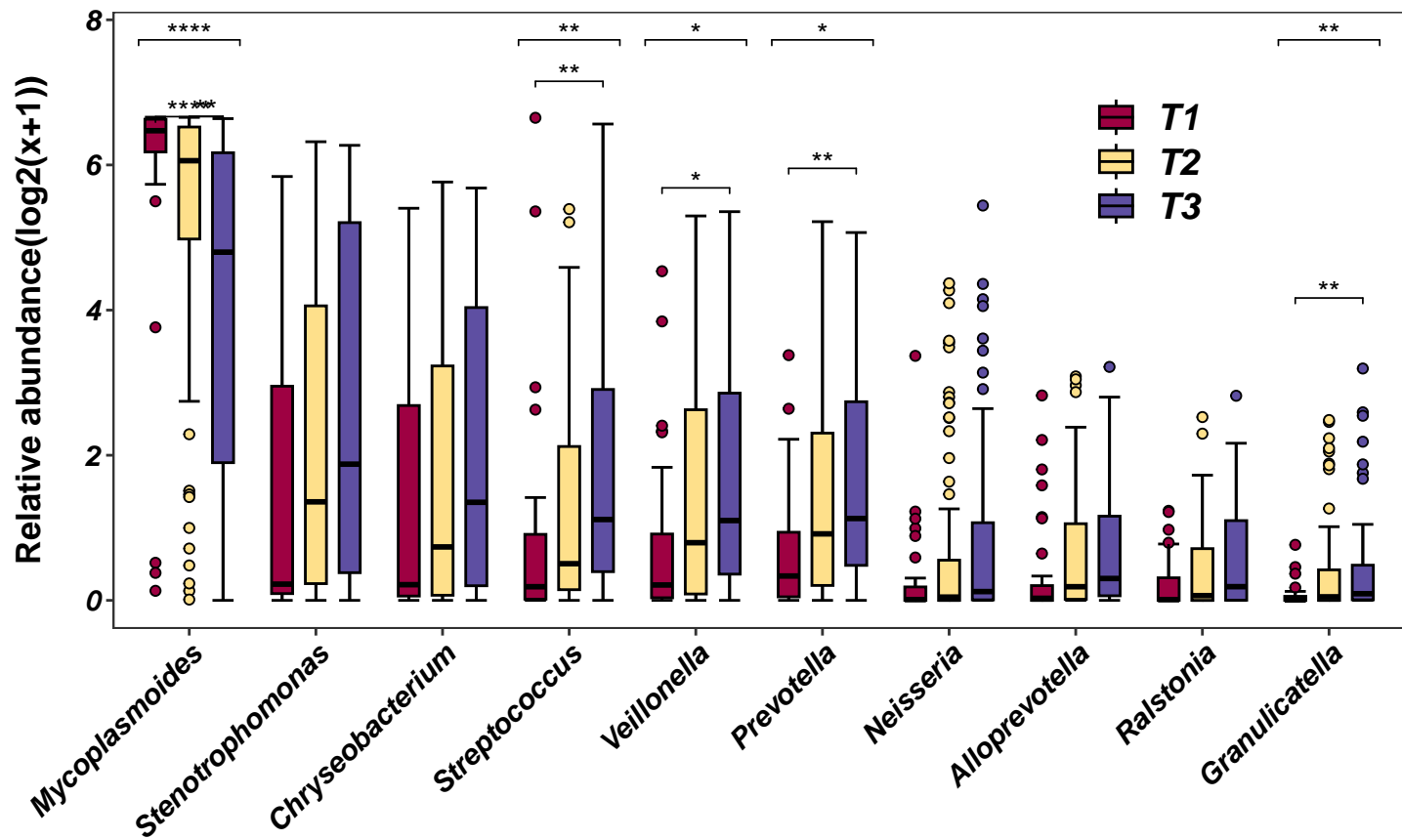

Supplement: Supplementary file 1 [file Presentation_1.pdf]
